# M3OT: A Multi-Drone Multi-Modality dataset for Multi-Object Tracking

**DOI:** 10.1038/s41597-025-06204-0

**Published:** 2025-12-08

**Authors:** Zhihao Nie, Luyi Xue, Zhenyu Fang, Jinchang Ren, Yufeng Wei, Jiangbin Zheng

**Affiliations:** 1 School of Software, Northwestern Polytechnical University, Xi’an, 710072 China; 2 National Subsea Centre, Robert Gordon University, Aberdeen, AB10 7QB UK; 3 School of Computer Science and Technology, Northwestern Polytechnical University, Xi’an, 710072 China

**Keywords:** Databases, Scientific community

## Abstract

We provide a dataset for object detection and tracking in aerial imagery, namely “M3OT”. M3OT is a multi-modality vehicle detection and tracking dataset acquired by two Unmanned Aerial Vehicles (UAVs) in a high-altitude region, consisting both RGB and infrared thermal (IR) modalities, ranging from 100 m to 120 m. Owing to the high-altitude acquisition, the annotated objects in the dataset are predominantly small objects, which poses significant challenges for detection and tracking algorithms. The dataset comprises 21,580 frames extracted from 8-hour videos, including 10,790 paired RGB-IR images, with a total of 220,000 bounding boxes annotated across various environments such as suburban, urban, daytime, dusk, and night. To our knowledge, this is the first multi-drone multi-modality dataset designed for multiple object tracking. We evaluate state-of-the-art multiple object tracking algorithms on this dataset. The experimental results indicate that the M3OT dataset presents a challenging benchmark for multiple object tracking. We believe that the M3OT dataset can contribute to applications and research based on vehicle detection and tracking from a UAV perspective. The dataset is freely available at https://figshare.com/s/01fa8d1163f4e9a5a13a

## Background & Summary

**Fig. 1:**
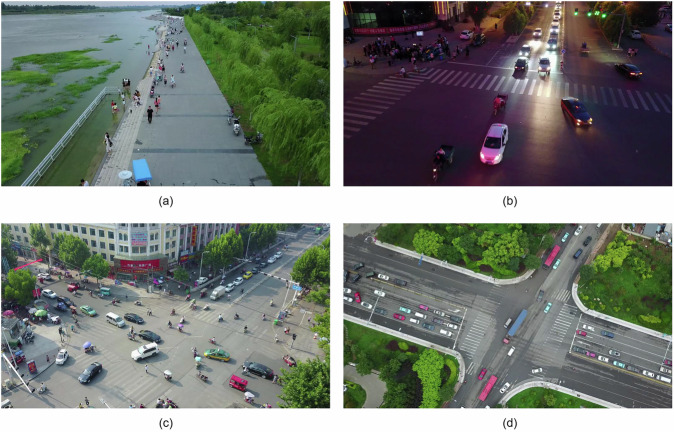
Sample images from UAV-captured scenes, highlighting challenges in object tracking. UAV images are affected by dynamic viewpoint changes and irregular object movements. These factors significantly increase the complexity of tracking tasks, because objects may shift unpredictably due to both UAV motion and target dynamics.

Unmanned Aerial Vehicles (UAVs) have been widely used in many fields, such as smart city construction^1^, environmental monitoring^2^, power system inspection^3^ and traffic management^4^. Compared with static cameras, UAV imaging can cover more expansive areas and more various viewpoints, which facilitates the detection and tracking of moving targets in rapidly changing environments. As shown in Fig. 1, UAV-captured images exhibit unique characteristics, such as dynamic viewpoint changes and irregular object movements, which significantly increase the complexity of tracking tasks.

To address these challenges, the research community has developed a variety of UAV-based datasets. Many of these are dedicated to object detection, such as CARPK^5^ for vehicle counting, AU-AIR^6^ for low-altitude multi-modal detection, the high-altitude thermal dataset HIT-UAV^7^, and TinyPerson^8^ for small-scale person detection. While these benchmarks are crucial for advancing object detection, they are generally designed for single-frame analysis. Consequently, they often lack the continuous, inter-frame identity annotations essential for developing and evaluating algorithms for multi-object tracking, which is a distinct and more complex task.

**Fig. 2:**
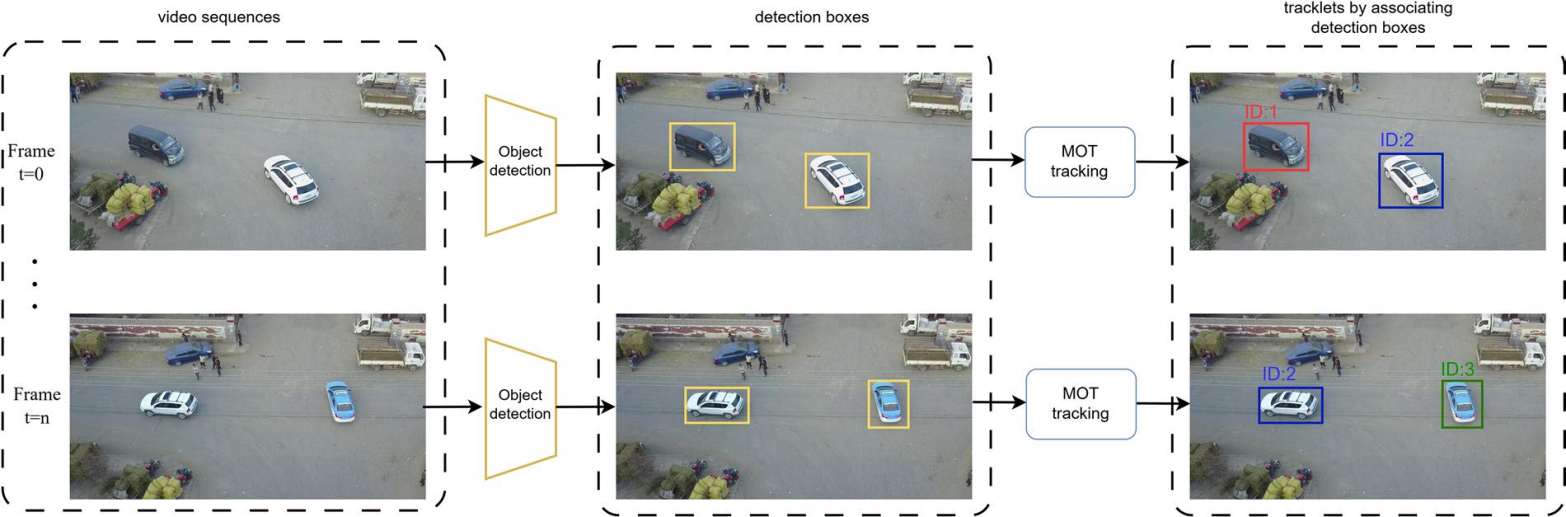
Detection-Based Tracking (DBT) framework: The DBT method starts with the use of an object detection algorithm to detect the location and category of objects in each frame. Following detection, a matching algorithm associates the objects with existing tracklets, maintaining continuity in tracking. Newly detected objects are assigned new tracklets(green box in the figure), while the tracklets of continually detected objects are updated across successive frames(blue box in the figure). When an object is no longer detected or moves out of the field of view, its trajectory is terminated(red box in the figure).

Multiple object tracking (MOT) aims to accurately track multiple objects across consecutive image frames, achieving by assigning each object a unique identity (ID) while maintaining the consistency of object identities. Most existing MOT approaches adhere to the Detection-Based Tracking (DBT) framework. As illustrated in Fig. 2, the DBT method begins by employing an object detection algorithm (e.g., Faster R-CNN^9^ or YOLO^10^) to identify the location and category of objects in each frame. After that, a matching algorithm is used to associate these detected objects with existing tracklets, thereby ensuring continuity in tracking. New objects that appear are assigned new tracklets, while tracklets for objects that are consistently detected are updated over successive frames. Conversely, tracklets are terminated when an object is no longer detected or exits the field of view.

With the rapid development of computer vision, several MOT datasets have been proposed for performance evaluation, such as MOTChallenge^11^ and DukeMTMC^12^. However, most of these datasets are collected from surveillance videos, which inherently possess certain limitations. For example, the annotated bounding box areas in these videos are predominantly larger than 1024 pixels, and the tracklets tend to follow linear motion patterns. These characteristics restrict the dataset’s ability to represent more complex tracking scenarios. Moreover, many popular MOT datasets, including MOT17^11^, MOT20^13^, and DanceTrack^14^, are designed exclusively for the RGB modality, making them inadequate for addressing challenges such as low-light conditions and other complex environmental scenarios. In low-light conditions, infrared images offer clearer object visibility compared to visible light images. Many studies have demonstrated that the integration of visible light and infrared thermal data can significantly enhance algorithm performance in tasks such as object detection and tracking. Leveraging the complementary information from both visible and infrared images can further improve tracking performance in complex scenarios. To support research in these areas, several benchmark datasets, such as RGBT210^15^, RGBT234^16^, and LasHeRare^17^, have been proposed for single object tracking tasks. However, these datasets are specifically designed for single-object tracking, limiting their applicability to multi-object tracking tasks.

To address the aforementioned limitations, researchers have also proposed other datasets for specific scenarios. For instance, the VisDrone^18^ dataset is an RGB visible light multi-object tracking dataset captured from an aerial perspective using drones, providing a valuable resource for multi-object tracking research in aerial photography. Additionally, the ASL-TID^19^ dataset is a single-object tracking dataset captured from an aerial thermal perspective, which offers crucial support for tracking people in adverse weather or low-light conditions. Although these datasets have made contributions in their respective fields, there is still a lack of a comprehensive dataset that can simultaneously cover multi-modality, multi-object, and complex motion patterns to fully advance research in this area.

Exploiting complementary information from multiple views can significantly enhance the robustness of tracking models, particularly in scenarios involving occluded objects. Consequently, multi-camera cooperative perception has emerged as a promising area of research, offering novel solutions for improving object detection and tracking in complex environments. Many multi-object, multi-camera datasets have been created, such as USC Campus^20^, PETS2009^21^, DukeMTMC^12^, CamNet^22^, and NLPRMCT^23^. Compared to a single UAV tracking task, using multiple drones can better combine information from different views, making tracking more reliable even in cases of occlusion. For multiple drone multiple object tracking tasks, MDMT^24^ is the first multiple drone multiple object tracking dataset, which contains RGB modality images taken by two drones.

Although many datasets have been introduced for MOT, there are still many challenges in this field:

- Limitations on small object tracking: As shown in Table 1, the bounding box areas of objects in the existing MOT datasets are all larger than 1024 pixels. According to the widely accepted definition of small objects in the MS COCO^25^ dataset, small objects are defined as those with a resolution smaller than 32 × 32 pixels (i.e., less than 1024 pixels), which limits the applicability of these datasets in small object tracking tasks.

**Table 1:** The record information of different datasets.

Dataset	Multi-view	Modality	Scenario	Resolution	Boxes Size	Class	Year
ASL-TID^19^	No	IR	Drone	1920 × 480	>1024	Multi-class	2014
DukeMTMC^12^	Yes	RGB	Surveillance	1920 × 1080	>1024	Person	2016
MOT17^11^	No	RGB	Surveillance	1920 × 1080	>1024	Person	2017
MOT20^13^	No	RGB	Surveillance	1920 × 1080	>1024	Person	2020
DroneVehicle^34^	No	RGB/IR	Drone	840 × 712	>1024	Vehicle	2021
VisDrone^18^	No	RGB	Drone	1920 × 1080	>1024	Multi-class	2021
MDMT^24^	Yes	RGB	Drone	1920 × 1080	>1024	Vehicle	2022
DanceTrack^14^	No	RGB	Surveillance	1280 × 720	>1024	Person	2022
MMptrack^35^	Yes	RGB	Surveillance	1920 × 1080	>1024	Person	2023
**M3OT(Ours)**^30^	**Yes**	**RGB/IR**	Drone	640 × 512	**<1024**	Vehicle	2025
- Limitations of single modality and single view: Many MOT datasets use only one sensor type or a single camera view, which has built-in limitations. For example, single modality systems often struggle in low-light conditions, making them less useful in dark environments. Likewise, single view systems have difficulty tracking objects when occlusions occur because overlapping objects can hide objects. Table 1 illustrates the information contained in various multi-object tracking datasets, providing a comprehensive overview of their limitations.

**Fig. 3:**
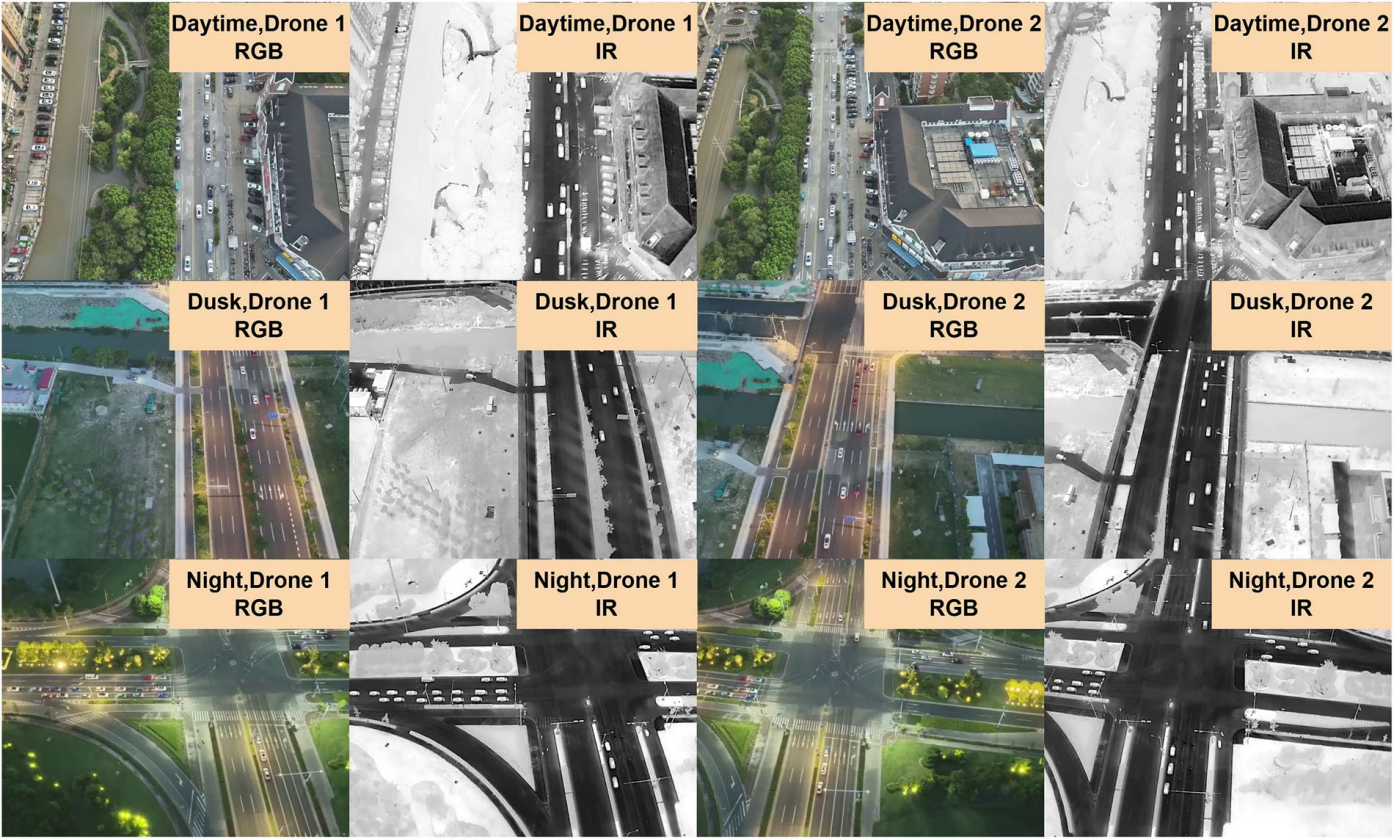
This figure presents a selection of sample images from the M3OT dataset, captured by two drones across three different environments: daytime, dusk, and night.

To overcome these challenges, we present the M3OT dataset, which includes multi-modality paired images captured by two UAVs. The proposed M3OT dataset can be applied to numerous tasks. For tasks involving detection and tracking across different modalities, M3OT provides both RGB and IR image sequences. Additionally, to facilitate research on multi-modality detection and tracking tasks, M3OT contains RGB-IR image pairs, with separate annotations for each modality. For multi-view multi-object tracking tasks, M3OT offers image pairs from different UAV perspectives within the same scene. As shown in Fig. 3, the M3OT dataset covers a variety of scenes, aiming to enrich the data distribution for different tasks.

The dataset comprises 21,580 frames extracted from 8 hours of video, including 10,790 paired RGB- IR images captured by two UAVs, along with 220,000 bounding boxes across diverse environments, such as suburban, urban, daytime, dusk, and nighttime settings. All frames were collected in public and desensitized. The dataset comprises 17260 training images, 2400 validation images, and 1920 test images. Unlike other RGB-IR multi-modality datasets, the M3OT dataset provides detailed annotations for both modalities, allowing corresponding annotation files to be used for training algorithm models. To evaluate the challenges of the M3OT dataset in the domain of multi-object tracking, we trained and tested advanced multi-object tracking algorithms, including ByteTrack^26^, OC-SORT^27^, ImprAssoc^28^, and Deep OC-SORT^29^. The results demonstrate that, compared to other multi-object tracking datasets, existing methods cannot achieve comparable results on the M3OT dataset as on other datasets. This is primarily due to the presence of small, non-linearly moving objects in M3OT, whereas other datasets typically contain larger, more linear-moving targets.

To the best of our knowledge, the M3OT dataset is the first publicly available multi-object tracking dataset designed for small object detection. M3OT has the significant potential to advance various research tasks, including: (1) small object detection and tracking, (2) multiple object detection and tracking in multi-modality images, (3) multiple object tracking across multiple viewpoints, and (4) multiple object tracking in multiple viewpoints, multi-modality settings.

## Methods

The video image sequences were captured using the DJI Mavic 3 T (https://enterprise.dji.com/cn/mavic-3-enterprise/specs) UAV platform. The detailed specifications of the cameras mounted on the UAV are shown in Table 2. The DJI Mavic 3 T is equipped with three cameras: a telephoto camera, a wide-angle camera, and an infrared thermal camera. The telephoto camera has a resolution of 4000 × 3000 pixels, while the infrared thermal camera has a resolution of 640 × 512 pixels. The cost of the DJI Mavic 3 T is approximately $4000.

**Table 2:** DJI Mavic3T Camera Parameters.

Camera Type	Telephoto Lens	Infrared Lens	Wide-Angle Lens
Actual Focal Length	29.85 mm	9.1 mm	4.4 mm
Photo Size	4000 × 3000 (4:3)	640 × 512 (4:3)	8000 × 6000
Effective Pixels	12 million	\	48 million
Sensor Type	1/2” CMOS	\	1/2” CMOS
Sensor Size	6.4 mm × 4.8 mm	7.68 mm × 6.144 mm	6.4 mm × 4.8 mm
35 mm Equivalent Focal Length	162 mm	40 mm	24 mm

The dataset generation pipeline consists of four stages: video capture, frame extraction and data cleaning, object annotation, and dataset creation.

### Video capture

The M3OT dataset was collected by two professional UAV operators, who carried out the dataset collection work in the airspace of 100 m–120 m. Apart from daylight scenes, we also collected data in low-light environments to reflect the complementary effect between cross-modality data. Thus, the M3OT dataset contains data from many different environments, which brings diversity to the training data of the model and can better cover the requirements of UAV multi-object tracking in real scenarios.

### Frame extraction and data cleaning

After data collection, low-quality video sequences such as blurred shots were discarded by manual selection. Meanwhile, frames from two modalities were also manually aligned, ensuring that the data collected by the two drones have temporal uniformity. In other words, each pair of video sequences has the same start and end time. In order to ensure the reliability of the data, we obtained clean unannotated data after manually checking all the sequences. We first perform distortion correction on all cleaned images. Since the attitudes of the drone were difficult to maintain absolute stable during the data collection, the cross-modality image pair captured by the two cameras will inevitably appear pixel misalignment. In the calibration stage, we perform affine transformation and region cropping on each RGB-IR image pair to ensure that most cross-modality image pairs are aligned. It is essential to note that when we use drones to capture data, we strictly comply with the laws and regulations of the location. Since the scale of the targets is very small, it is impossible to obtain identification information such as faces and vehicle plates. After carefully checking, we confirm that all data in our dataset will not reveal any personal information.

### Object annotation

We use CVAT (https://www.cvat.ai/) annotation software to annotate video sequences that have been manually selected. The annotation work is assigned by four researchers, expert in the field of computer vision, using the management tools of CVAT annotation software. After that, a final verifier is assigned to ensure that all annotations are accurate. We set a different ID number for each target and checked to make sure there were no duplicate ID labels. Finally, the annotation results are saved as “gt.txt” files in the MOT format. The gt.txt annotation file stores information about the detection boxes in the image sequence, with each row representing a detected object. The format is as follows: (frame_id, track_id, x, y, w, h, ‘not ignored’, class_id, conf). Here, “frame_id” indicates the frame number, “track_id” represents the ID assigned to the detection box, and (x, y, w, h) correspond to the top-left coordinates and dimensions (width and height) of the bounding box. The field ‘not ignored’ specifies that the object is not ignored, “class_id” denotes the ID of the object’s class, and conf represents the confidence score.

## Data Records

The data is available at figshare^30^.

### Folder structure and recording format

We provide users with two types of annotation files: the GT file, based on the MOTChallenge dataset format, and the JSON file, based on the MS COCO dataset format. The GT file is intended for multi-object tracking tasks, while the JSON file is designed for object detection tasks. The JSON files are stored in the ‘Annotations’ folder of the dataset, whereas the GT files are located in the ‘gt’ folder within each image sequence folder.

### Properties

**Fig. 4:**
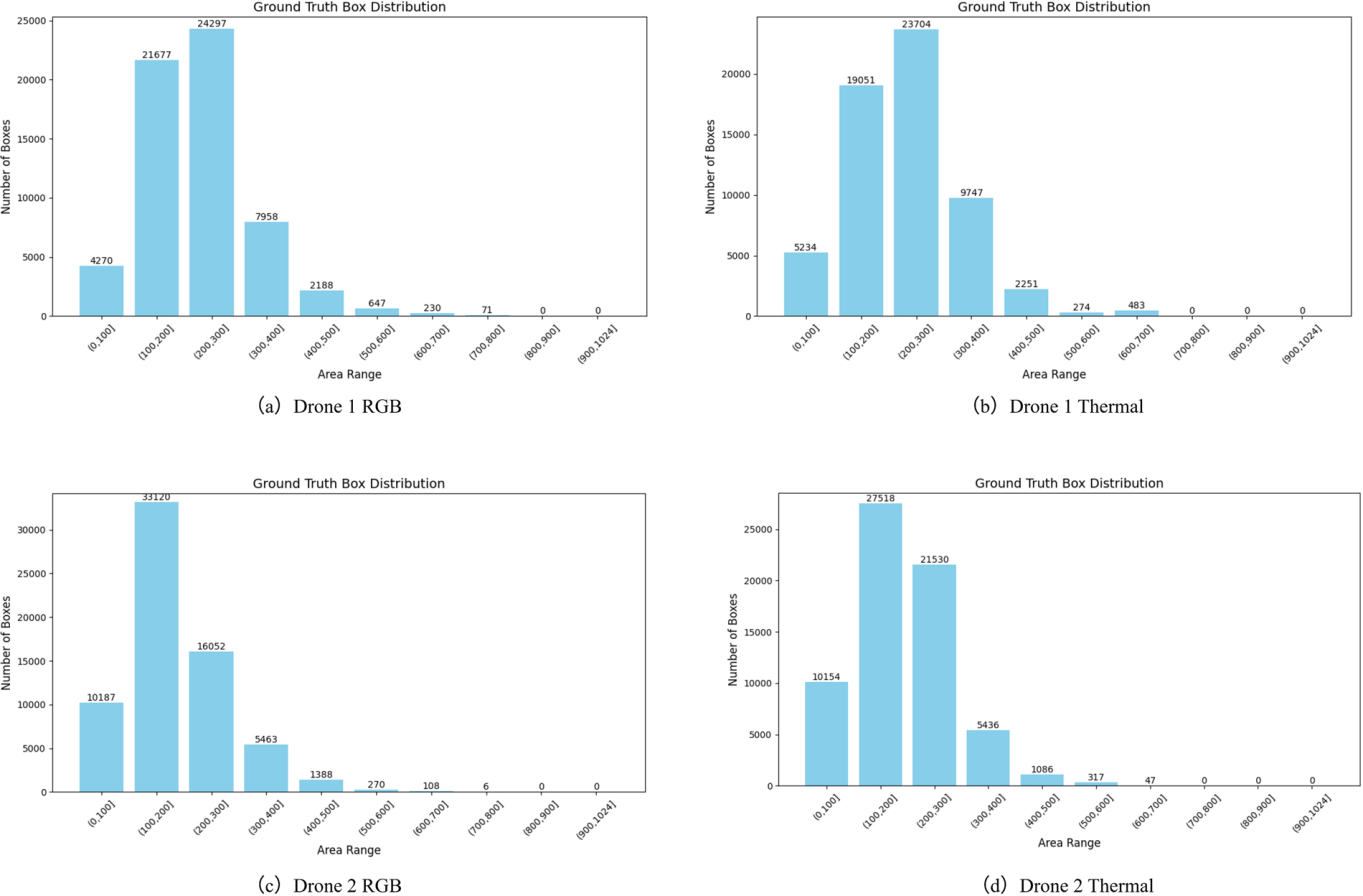
The distribution of ground truth boxes in the M3OT dataset is shown as follows: (**a**) distribution of ground truth boxes in the RGB modality data captured by Drone 1, (**b**) distribution of ground truth boxes in the infrared thermal modality data captured by Drone 1, (**c**) distribution of ground truth boxes in the RGB modality data captured by Drone 2, and (**d**) distribution of ground truth boxes in the infrared thermal modality data captured by Drone 2.

In the field of object detection, a small object typically refers to an object that occupies a relatively small area in an image. The definition of a small object can vary depending on the context. For example, in the MS COCO dataset, small objects are defined as those with a resolution smaller than 32 × 32 pixels. The M3OT dataset also follows this standard for defining small objects. As shown in the Table 1, most objects in mainstream benchmark datasets for multi-object tracking do not meet the criteria for small objects, as object sizes are generally large. However, in the M3OT dataset, we have specifically annotated small objects to ensure the dataset includes a substantial number of objects with sizes below 1024 pixels. To provide a clearer view of the distribution of these small objects, we categorized and counted object frames across different size intervals in the dataset, calculating the proportion of object frames in each interval. As shown in Table 3, for data collected by Drone 1 in RGB modality, the distribution is as follows: The number of object frames with an area in the range of (0, 64] pixels is 709, which accounts for 1.16% of the total. The number of object frames with an area between (64, 256] pixels is 10,586, representing 17.26%. For object frames with an area between (144, 256] pixels, the count is 26,261, comprising 42.81%. Finally, the number of object frames with an area in the range of (256, 1024] pixels is 23,782, accounting for 38.77%. Figure 4 presents a more detailed distribution of the ground truth boxes. The area of ground truth boxes in the M3OT dataset primarily ranges between 100 and 400 pixels. Compared to other MOT datasets, M3OT focuses on small object tracking, thus filling the gap in training and evaluation benchmarks for small object tracking tasks.

**Table 3:** Bounding box size distribution in RGB and infrared thermal modalities across various drones in M3OT dataset, with sizes ranging from 0–64 to 256–1024 pixels.

Dataset	(0,64]	(64,144]	(144,256]	(256,1024]	Total
Drone1 RGB	709(1.16%)	10586(17.26%)	26261(42.81%)	23782(38.77%)	61338(100%)
Drone1 Thermal	1160(1.91%)	10002(16.46%)	22560(37.14%)	27022(44.49%)	60744(100%)
Drone2 RGB	1165(1.75%)	22237(33.39%)	26879(40.36%)	16313(24.50%)	66594(100%)
Drone1 Thermal	1631(2.47%)	16915(25.59%)	28726(43.47%)	18815(28.47%)	66088(100%)

All bounding boxes in M3OT are less than 1024 pixels in size.

## Technical Validation

### Experimental settings

We trained the YOLOv8 object detection algorithm separately on four subsets of the M3OT dataset (as shown in Fig. 4) and validated it using four state-of-the-art multi-object tracking algorithms. Each subset consists of 4,315 training images, 600 validation images, and 480 test images. The hardware is a single NVIDIA GeForce RTX 4090 GPU with Intel(R) Core(TM) i9-14900K CPU. We utilized the well-established Ultralytics framework (https://github.com/ultralytics/ultralytics), one of the most mature implementations of the YOLO algorithm, with the YOLOv8-n pre-trained model obtained from the official source. During training, the number of epochs was set to 300, the batch size was 32, and the Adam optimizer was employed. The learning rate was set to 0.001, and weight decay to 0.0005, with a warm-up ratio of 0.001. The weight decay and momentum were configured to 0.0005 and 0.937, respectively. For the tracker component, we selected the mature Box-MOT framework (https://github.com/mikel-brostrom/boxmot), which integrates a series of multi-object trackers. Four state-of-the-art trackers within the framework were chosen to evaluate our dataset. The tracking results generated by the trackers were analyzed using the TrackEval (https://github.com/JonathonLuiten/TrackEval) evaluation tool, a standardized tool for multi-object tracking performance assessment.

### Evaluation metrics

To more effectively evaluate the tracking performance of the algorithms on the M3OT dataset, we adopt several key evaluation metrics: MOTA (Multiple Object Tracking Accuracy)^31^, IDF1 (Identification F1 Score)^32^, and HOTA (Higher Order Tracking Accuracy)^33^. Below are the definitions of the three evaluation metrics, and the details will be offered in the supplementary material.

#### MOTA

This metric measures how well the tracker detects objects and predicts tracklets without taking precision into account.

#### IDF1

The ratio of correctly identified detection over the average number of ground-truth and computed detections.

#### HOTA

HOTA measures how well the tracklets of matching detections align, and averages this over all matching detections, while also penalizing detections that don’t match.

### Experimental analysis

Table 4 shows that tracking results of different object trackers on the M3OT dataset under various modalities and viewpoints. By comparing the tracking results across different modal data, the same multi-object tracking algorithm (e.g., ByteTrack) was trained using only RGB modal data, yielding a HOTA of 37.7% (Drone 1) and 36.0% (Drone 2), IDF1 of 59.0% (Drone 1) and 54.8% (Drone 2), and a MOTA of 38.9% (Drone 1) and 36.4% (Drone 2) on the validation set sequence. In contrast, when trained using only infrared thermal modal data, the HOTA is 34.9% (Drone 1) and 43.5% (Drone 2), with IDF1 scores of 62.1% (Drone 1) and 62.8% (Drone 2), and a MOTA of 43.6% (Drone 1) and 51.2% (Drone 2). The experimental results in Table 4 reveal the following findings:

- Under the infrared thermal modality, the MOTA and IDF1 metrics of all four trackers outperform those under the RGB modality.
- The infrared thermal modality effectively handles challenging scenarios, such as nighttime and low-light conditions, enabling robust multi-object tracking (MOT) in these environments.

**Table 4:** Tracking performance of different object trackers on the M30T dataset, across diverse modalities and viewpoints.

UAV	Tracker	Split	RGB			Thermal			Overall		
			HOTA↑	IDF1↑	MOTA↑	HOTA↑	IDF1↑	MOTA↑	HOTA↑	IDF1↑	MOTA↑
Drone 1	ByteTrack	val	37.7	59.0	38.9	34.9	62.1	43.6	36.3	60.6	41.3
	OC-SORT	val	36.8	54.3	34.8	31.8	55.3	35.3	34.3	54.8	35.1
	Deep OC-SORT	val	31.1	47.7	27.8	31.7	55.8	36.2	31.4	51.8	32.0
	ImprAsso	val	29.6	40.1	28.8	28.2	43.0	40.0	28.9	41.6	34.4
Drone 2	ByteTrack	val	36.0	54.8	36.4	43.5	62.8	51.2	39.8	58.8	43.8
	OC-SORT	val	33.9	49.7	31.8	37.7	50.1	41.9	35.8	49.9	36.9
	Deep OC-SORT	val	32.1	47.1	28.5	38.3	54.2	44.7	35.2	50.7	36.6
	ImprAsso	val	28.9	37.1	29.6	40.1	48.3	48.7	34.5	42.7	39.2

Table 5 presents the evaluation results of the same four object trackers on the MOT17, MOT20, and M3OT datasets. The evaluation results lead to the following insights:

- The four object trackers demonstrate higher performance on MOT17 and MOT20 datasets than on the M3OT dataset.
- Unlike most conventional MOT datasets, which primarily focus on tracking larger objects with relatively straightforward motion patterns, the M3OT dataset is specifically designed to address the challenges of small-object tracking. This focus introduces higher levels of complexity, as small objects often have non-linear tracklets and occupy minimal pixel areas, making tracking inherently more difficult.
- Current object trackers exhibit challenges when tracking small objects within the M3OT dataset. This limitation highlights the dataset’s potential to drive advancements in the study of small-object tracking.

**Table 5:** Evaluation of object tracking methods on MOT17, MOT20, and M30T datasets based on HOTA, IDF1, and MOTA scores.

Dataset	Tracker	RGB		
		HOTA↑	IDF1↑	MOTA↑
MOT17	ByteTrack	63.1	77.3	80.3
	OC-SORT	63.2	77.5	78.0
	Deep OC-SORT	64.9	80.6	79.4
	ImprAsso	66.4	82.1	82.2
MOT20	ByteTrack	61.3	75.2	77.8
	OC-SORT	62.1	75.9	75.5
	Deep OC-SORT	63.9	79.2	75.6
	ImprAsso	64.6	78.8	78.6
M3OT(Ours)	ByteTrack	36.9	56.9	37.7
	OC-SORT	35.4	52.0	33.3
	Deep OC-SORT	31.6	47.4	28.2
	ImprAsso	29.3	38.6	29.2

**Fig. 5:**
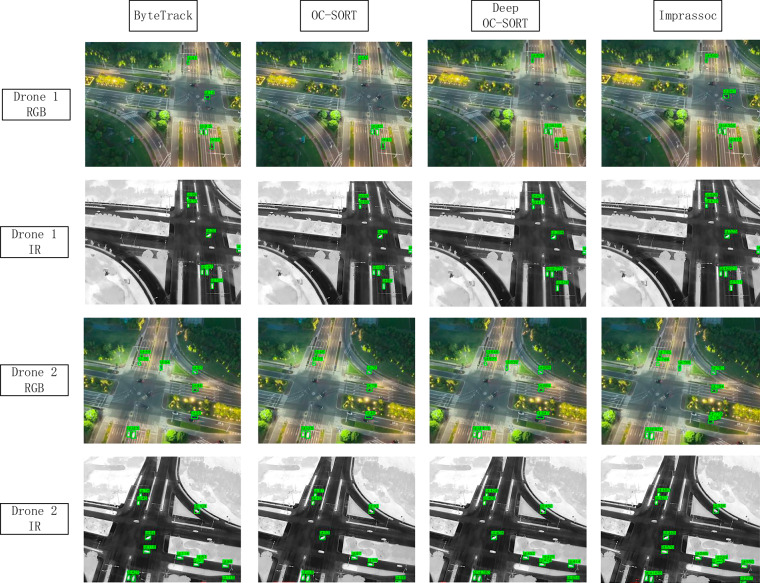
The sample tracking results for the four object trackers.

Figure 5 illustrates the sample tracking results for the four object trackers on the M3OT dataset. In the field of small-object tracking, the development of lightweight and high-performance multi-object tracking algorithms is a promising research direction. The M3OT dataset supports multiple tasks, including cross-modality or single-modality object detection and tracking, multi-view or single-view object detection and tracking, as well as small-object detection and tracking. We hope that the M3OT dataset will drive research related to drone-based small-object detection and tracking.

## Usage Notes

The M3OT dataset is available on figshare^30^, where users can download it to train object detection and tracking algorithms. We provide label files in both GT and MS COCO formats, making M3OT easy to use.

The M3OT dataset was collected across different time periods, including daytime, dusk, and nighttime, as well as in diverse environments such as urban and suburban areas. This diversity enhances the dataset’s ability to support the training of object detection and tracking models, improving their adaptability to real-world UAV multi-object tracking tasks. Researchers can use M3OT to train models to research the application range of multi-modality (RGB-IR) fusion and multi-view cooperation in different tracking tasks. Additionally, the trained models have the potential to be employed in UAV-based intelligent transportation monitoring to analyze traffic flow and critical infrastructure surveillance during nighttime to evaluate their feasibility and robustness.

## Data Availability

The data processing code is available in the tools folder of https://github.com/M3OT/M3OT. The code is written in Python. The functions of the tools are as follows: (1) The tools/M3OT2coco.py is to generate the label with the COCO format to help users train the algorithm. (2) The tools/convert_M3OT_to_yolo.py is to generate the label files with the YOLO format to help users train the YOLO algorithm.
